# Periodontal disease in a patient receiving Bevacizumab: a case report

**DOI:** 10.1186/1752-1947-2-47

**Published:** 2008-02-13

**Authors:** Dorothy M Gujral, Sanjeev Bhattacharyya, Peter Hargreaves, Gary W Middleton

**Affiliations:** 1 St Lukes Cancer Centre, Royal Surrey County Hospital, Guildford, UK

## Abstract

**Introduction:**

Bevacizumab is a monoclonal antibody that inhibits the action of vascular endothelial growth factor (VEGF) thereby acting as an angiogenesis inhibitor. As a result, supply of oxygen and nutrients to tissues is impaired and tumour cell growth is reduced. Reported side effects due to bevacizumab are hypertension and increased risk of bleeding. Bowel perforation has also been reported. Periodontal disease in patients on bevacizumab therapy has not been reported before.

**Case Presentation:**

We report a case of a forty-three year old woman who developed periodontitis whilst receiving bevacizumab for lung cancer. The periodontal disease remained stable on discontinuation of the drug.

**Conclusion:**

Further investigations are needed to determine the mechanism for bevacizumab-induced periodontal disease.

## Introduction

Bevacizumab is a monoclonal antibody that inhibits the action of vascular endothelial growth factor (VEGF) thereby acting as an angiogenesis inhibitor and preventing the formation of new blood vessels, including those that surround and supply cancer cells. As a result, supply of oxygen and nutrients to tissues is impaired and tumour cell growth is reduced. Cancerous tumours may become slower growing or even smaller.

It is this property that has found non-oncological uses for bevacizumab as its anti-angiogenesis effect has been useful in the treatment of proliferative (neovascular) eye diseases, particularly age-related macular degeneration [1-4].

Reported side effects due to bevacizumab are hypertension and increased risk of bleeding. Bowel perforation has also been reported [5-8].

Periodontal diseases range from simple gum inflammation to serious disease that results in major damage to the soft tissue and bone that support the teeth. Risk factors for the development of periodontal disease are smoking, hormonal changes in women, poor nutrition resulting in deficiencies in calcium and certain vitamins (especially vitamins B and C), diabetes, gingivitis, stress, immunosuppressive illnesses, genetic susceptibility and certain medications (including chemotherapy drugs and those that reduce the production of saliva such as antidepressants, antihistamines, antiepileptics, and calcium channel blockers). [9,10].

Previous studies have shown increased levels of VEGF in gingival and gingival crevicular fluid in patients with periodontitis, in keeping with an inflammatory process induced by periodontopathogens, with neovascularisation causing swelling and oedema [11,12]. It is possible that VEGF levels are high in an attempt to stimulate angiogenesis and facilitate healing. Regeneration of periodontal structures lost during periodontal disease is regulated by, among other things, interactions between cells and growth factors.

## Case Presentation

A 43 year old lady presented in 2005 with wheeze, shortness of breath and pain in the right back. She had no other symptoms of note and was an ex-smoker. The patient had had an ovarian cystectomy in 2004 which was complicated by fistula formation requiring several laparotomies.

At bronchoscopy, obstruction by a large right middle lobe tumour was noted and biopsy confirmed an adenocarcinoma. Subsequent PET-CT scanning revealed extensive soft tissue abnormalities in the right paravertebral region posteriorly and lymphadenopathy in the subcarinal, contralateral and pretracheal regions. The final staging was T2N3M0 (IIIB).

The patient was enrolled in the AVAiL trial (AVAstin In Lung cancer – Trial No. BO17704) – a study in which the primary objective is to evaluate safety and efficacy of two doses of bevacizumab in combination with gemcitabine and cisplatin and determine the optimal dose of bevacizumab. The trial is a randomised, double-blind, multicentre, 2-stage, phase III study of bevacizumab and gemcitabine/cisplatin versus placebo and gemcitabine/cisplatin in patients with advanced or recurrent non-small cell lung carcinoma who have not received prior chemotherapy. The patient was randomised to receive gemcitabine/cisplatin and bevacizumab on the maintenance arm.

**Figure 1:**
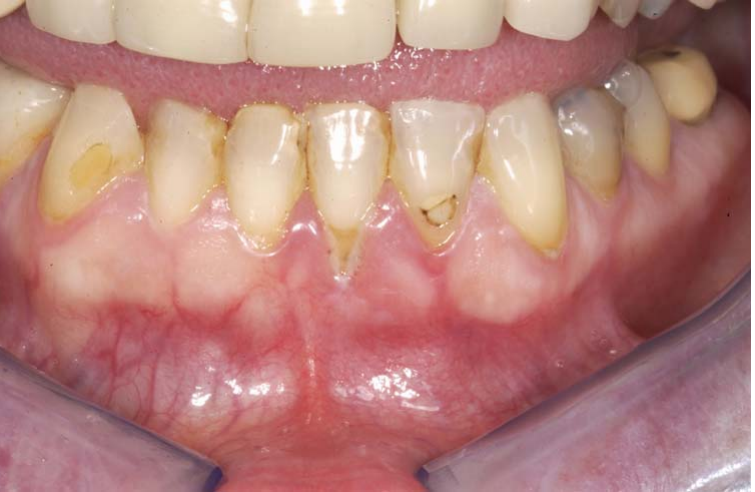
Periodontal disease noted at cycle 6 of bevacizumab (18 weeks into treatment).

Five cycles of treatment (carboplatin was substituted for cisplatin at cycle 3 due to toxicity) were completed in March 2006 with partial response. At cycle 6 (eighteen weeks into treatment), marked gum recession was noted (fig 1). The patient was then unblinded and found to be on the continuation arm of bevacizumab. Treatment was continued with no interventions and the patient remained entirely asymptomatic.

**Figure 2:**
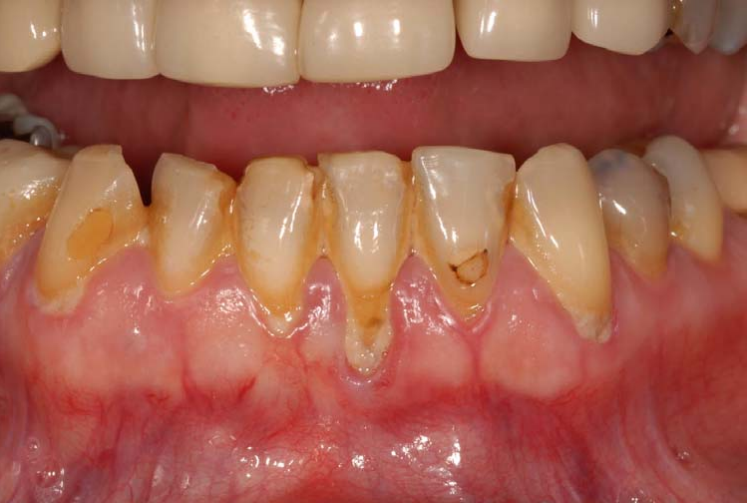
Worsening periodontal disease at cycle 18 of bevacizumab (10 months into treatment).

In September 2006 (ten months after commencing treatment), the patient was noted to have worsening periodontal disease (fig 2). She had completed 18 cycles of bevacizumab at this stage. The patient completed treatment in December 2006 and the periodontal disease has since remained stable.

This case is, to the best of our knowledge, the first reported of a patient developing periodontal disease whilst receiving bevacizumab. Although it would be difficult to exclude all other risk factors in this patient, the onset of periodontal disease on commencement of bevacizumab and the fact that the disease remained stable on discontinuation of the drug points to this as the cause.

## Conclusion

In this case, one might expect low plasma levels of VEGF with the use of bevacizumab. It is possible that, as an angiogenesis inhibitor, bevacizumab prevents the formation of new blood vessels, resulting in a lack of supply of oxygen and nutrients to the tissues. This may result in ischaemia and necrosis (consequently inhibiting reversal of the process and impairing wound healing). Further investigations are therefore needed to determine the mechanism for bevacizumab-induced periodontal disease.

## Competing interests

The author(s) declare that they have no competing interests.

## Authors' contributions

DG drafted the manuscript, DG and SB reviewed the literature. DG, PH and GM were involved in the patient's care and follow-up.

All authors read and approved the final manuscript.

## Consent

Written consent was obtained from the patient for publication of this case report and accompanying images. A copy of the written consent is available for review by the Editor-in-Chief of this journal.
